# Single Cell Bottlenecks in the Pathogenesis of *Streptococcus pneumoniae*

**DOI:** 10.1371/journal.ppat.1005887

**Published:** 2016-10-12

**Authors:** Masamitsu Kono, M. Ammar Zafar, Marisol Zuniga, Aoife M. Roche, Shigeto Hamaguchi, Jeffrey N. Weiser

**Affiliations:** 1 Department of Microbiology, New York University, New York, New York, United States of America; 2 Department of Otolaryngology-Head and Neck Surgery, Wakayama Medical University, Wakayama, Japan; 3 Department of Microbiology, University of Pennsylvania, Philadelphia, Pennsylvania, United States of America; Boston Children's Hospital, UNITED STATES

## Abstract

Herein, we studied a virulent isolate of the leading bacterial pathogen *Streptococcus pneumoniae* in an infant mouse model of colonization, disease and transmission, both with and without influenza A (IAV) co-infection. To identify vulnerable points in the multiple steps involved in pneumococcal pathogenesis, this model was utilized for a comprehensive analysis of population bottlenecks. Our findings reveal that in the setting of IAV co-infection the organism must pass through single cell bottlenecks during bloodstream invasion from the nasopharynx within the host and in transmission between hosts. Passage through these bottlenecks was not associated with genetic adaptation by the pathogen. The bottleneck in transmission occurred between bacterial exit from one host and establishment in another explaining why the number of shed organisms in secretions is critical to overcoming it. These observations demonstrate how viral infection, and TLR-dependent innate immune responses it stimulates and that are required to control it, drive bacterial contagion.

## Author Summary

Many discrete steps are involved in the progression of infectious diseases. Bottlenecks represent key points where the population size/genetic diversity is at a minimum and the pathogen is most vulnerable to intervention strategies. Our study used an infant mouse model for a comprehensive analysis of bottlenecks in infection by the major pathogen *Streptococcus pneumoniae*. In our model, we also considered influenza A virus, a clinically important and common co-infection. The main findings reveal i) a single cell bottleneck during host-to-host transmission and ii) the bottleneck in transmission occurs during events between bacterial exit from one host and establishment in another host. We manipulated innate immune responses involved in viral control and inflammation to show that viral co-infection allows the bottleneck in transmission to be overcome by increasing bacterial exit. Finally, we demonstrate that a specific host response stimulated by influenza A is sufficient to recapitulate effects of viral co-infection. Thus, our study identifies key vulnerable stages during *S*. *pneumoniae* infection and provides mechanistic understanding for how viral infection promotes bacterial contagion.

## Introduction

The pathogenesis of microbial diseases generally involves multiple stages (entry, establishment, invasion, exit) that often begin with the colonization of host surfaces. For organisms without an environmental reservoir, their continued success requires proliferation within their obligate host and transmission to new susceptible hosts. The induction of disease, which usually results from a combination of impaired host defense and microbial virulence attributes, may benefit the organism if it increases proliferation and/or transmission. An example of an organism with a predominantly commensal lifestyle that also is a leading cause of disease is *Streptococcus pneumoniae* (the pneumococcus) [1]. Pneumococci serially and sequentially colonize the mucosal surface of the human nasopharynx asymptomatically beginning in early childhood (the carrier state). Transmission occurs from carriers to non-carriers and is most frequent in settings of close contact, such as among siblings or in daycare centers, and results from direct or indirect exposure to respiratory secretions [2][3]. Disease occurs when the organism transits to normally sterile sites within the respiratory tract to the middle ear cavity or lungs. The organism may also gain access to the bloodstream from the nasopharynx or sites of localized disease to cause systemic infection. Because of high rates of carriage and these complications, the pneumococcus is a leading cause of otitis media, pneumonia, and sepsis [1][4]. An additional consideration is that recent upper respiratory viral infection, particularly with influenza, increases rates of carriage and is a major risk factor for all pneumococcal diseases [5][6][7].

Pneumococcal infection has been partially controlled through immunization. The most well established effect of vaccine-induced immunity in adults is protection of the individual from pneumococcal bacteremia [8][9]. More recently, widespread immunization of children, which blocks the acquisition of colonization, has led to lower rates of transmission within the community and protection of unvaccinated populations (‘herd immunity’) [10][11]. Together these clinical observations suggest that it is possible to impact pneumococcal disease at discrete steps in its pathogenesis.

In order to better understand the mechanisms responsible for protection, we sought to identify the steps during infection where pneumococci must pass through a population bottleneck(s)–a sharp reduction in the size of the population due to environmental constraints [12][13]. A bottleneck would occur, for instance, if only a fraction of infecting organisms are able to pass through a host barrier or evade a local host defense. Alternatively, a bottleneck could result from microbial competition or a requirement for increased fitness via genetic adaptation (within-host evolution) to meet different challenges in the host. Tight bottlenecks would be attractive ‘weak points’ for intervention strategies since there would be relatively few organisms to target. Also, by targeting the stages when the bacterial population size is most restricted, there is less opportunity for selection among the phenotypically and genetically diverse population that characterizes the pneumococcus.

Since there are no tractable experimental models to study steps beyond colonization in the natural host, we examined these in an animal model. Our study employed an infant mouse model that recapitulates many of the key features of pneumococcal pathogenesis. These include increased susceptibility early in life, occurrence of localized disease in normally sterile sites within the respiratory tract (otitis media) and invasive infection (bacteremia) following colonization, and close contact leading to increased host-to-host transmission.

## Results

### Influenza A co-infection enhances colonization, otitis media and transmission

**Fig 1:**
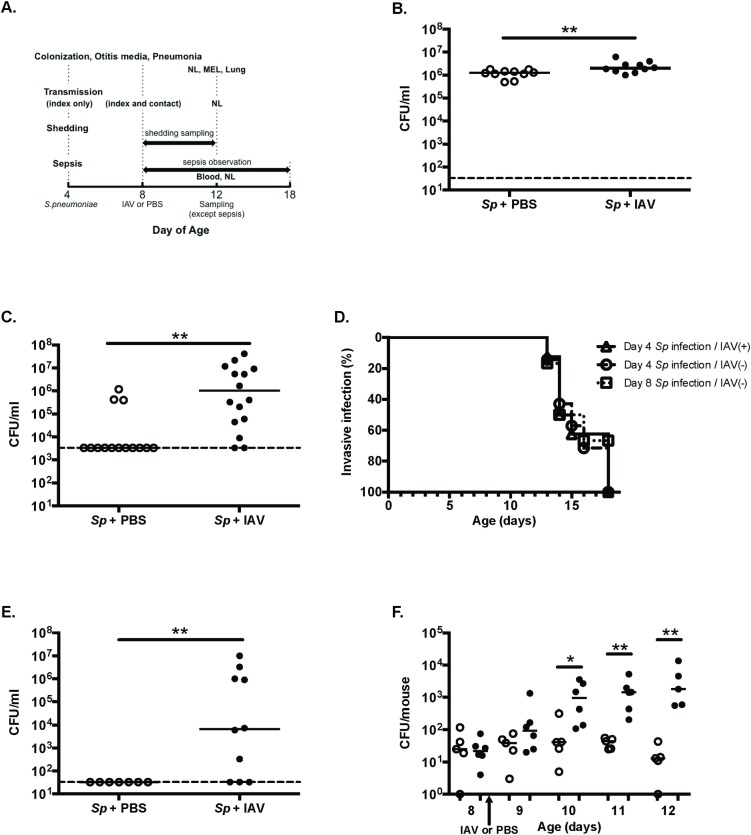
Infant mouse model for co-infection of *S*.*pneumoniae* and influenza virus. **A.** Schematic of the experimental schedule. On day 4 of age, pups were intranasally inoculated with a serotype 6A clinical isolate of *S*.*pneumoniae*. At age 8 days, the pups were inoculated with influenza A virus (IAV strain x31) or PBS. On day 12 of age, the pups were sacrificed and samples listed (NL, nasal lavage. MEL, middle ear lavage. Lung, lung homogenate) were collected. The following changes to the schematic described above were made. For the transmission experiments, one pup (index) was infected with *S*.*pneumoniae* and returned to the littermates (contacts). For the sepsis experiments, pups were not sacrificed at day of age 12 and monitored until showing sign of sepsis or euthanized at day of age 18 to obtain blood cultures. For the shedding experiments, daily cultures of secretions were obtained from day of age 8–12. **B, C, E, F.** Comparisons between the *S*.*pneumoniae* (*Sp*) and IAV co-infection group (black circle) and the *S*. *pneumoniae* plus PBS control infection group (open circle) and in each infection model. Each dot represents a single mouse. Mann-Whitney U test was used for the statistical analyses. *p<0.05 and **p<0.01 respectively. The dashed line indicates the detection limit. **B.** Colonization assessed by the density of pneumococci in nasal lavages. **C.** Otitis media assessed by the density of pneumococci in middle ear lavages. **D.** Time course of septic or sustained bacteremic infection. Pups were colonized with *S*. *pneumoniae* at age 4 days with (solid line, triangle, n = 8) or without IAV (dashed line, circle, n = 7) or at age 8 days without IAV (dotted line, square, n = 6). **E.** Transmission assessed by the density of pneumococci in nasal lavages of contact mice. **F.** Shedding assessed by the number of bacteria in secretions on the day of age indicated.

A type 6A clinical isolate of *S*. *pneumoniae* was tested in a previously described infant mouse model with intranasal (IN) challenge at age 4 days [14][15]. An advantage of this model is that infant mice are susceptible to a low colonizing dose [16]. To examine the effect of influenza A virus (IAV) co-infection, the mouse-adapted strain x31 was inoculated at age 8 days (schematically represented in **Fig 1A**). The density of colonization was determined in nasal lavages obtained at age 12 days. All pups were heavily colonized and mice co-infected with IAV showed a significantly higher burden of organisms compared to controls (**Fig 1B**).

Otitis media was assessed in lavages of the middle ear cavities obtained through the tympanic membrane on day of age 12 using microsurgery. In pilot experiments, we confirmed that the presence of pneumococci in middle ear lavages correlated with an acute inflammatory response as indicated by an increase in numbers of neutrophils (CD45^+^, CD11b^+^, Ly6G^+^ cells) detected by flow cytometry (data not shown). Pneumococci were detected in 87% of middle ear samples among the IAV co-infected group compared to 21% of the PBS control group (**Fig 1C**).

To detect systemic infection after intranasal challenge, the observation period was extended and beginning at age 13 days mice developed signs of sepsis. Pneumococcal bacteremia was confirmed by blood culture in moribund mice or in mice surviving until the conclusion of the observation period at 18 days of age. Sepsis was not detected prior to age 12 days. All mice eventually developed invasive infection and there was no statistical difference in the time course of sepsis (or bacteremia) between IAV co-infected and PBS control animals (*p* = 0.85) (**Fig 1D**).

To examine pup-to-pup transmission, one 4-day old pup was selected from the litter (index mouse) and colonized with *S*.*pneumoniae*. On day 8, both the index and all other pups (contact mice) were inoculated with IAV or PBS. The transmission experiment followed the previously described protocol except it was terminated at age 12 days [14]. A shorter observation period was used to minimize transmission among contact mice, since once acquiring the organism a contact mouse could become a source of transmission. It was not possible to abbreviate this observation period further since in pilot experiments at time points prior to age 12 days transmission was unusual. Under the conditions tested, 70% of contact mice became colonized by age 12 days. In contrast, for the control group where all pups were inoculated with PBS instead of IAV at age 8 days, no transmission events were observed (**Fig 1E**).

Previously, we reported that transmission was proportional to the level of pneumococcal shedding from the index pup [14]. To evaluate the impact of IAV on pneumococcal shedding, pups were treated as index mice (colonized at day of age 4 and infected with IAV or PBS at age 8 days) and the number of shed bacteria measured daily from day 8 to 12. IAV co-infection increased the mean shedding of pneumococci on days 10–12 by ~10–50 fold (**Fig 1F**).

Thus, following intranasal challenge with this single isolate, infant mice became colonized, developed respiratory tract (otitis media) and invasive infection (bacteremia/sepsis), and transmitted the organism among the litter. Influenza A significantly increased the burden of colonization and risk of otitis media. Additionally, influenza A co-infection was permissive for transmission–an effect that correlated with enhanced shedding.

### Lack of a tight population bottleneck in colonization and otitis media

To assess bacterial population bottlenecks, three mutants, each tagged with a different antibiotic resistance marker inserted into the gene encoding the pneumococcal IgA1 protease were constructed. This gene was selected because mice do not express a protease susceptible form of immunoglobulin and it was previously shown that *iga* mutants show no defect in murine infection [17]. There was no difference in the in vitro growth characteristics of the three strains alone or in combination (S1A Fig and S2B Fig). When inoculated equally in combination their overall ability to colonize the infant mouse nasopharynx was equivalent (S2A Fig) and indistinguishable from the parent strain. The size of population bottlenecks was estimated by examining the distribution of isogenic mutants passing through each stage in pathogenesis with or without IAV co-infection.

**Fig 2:**
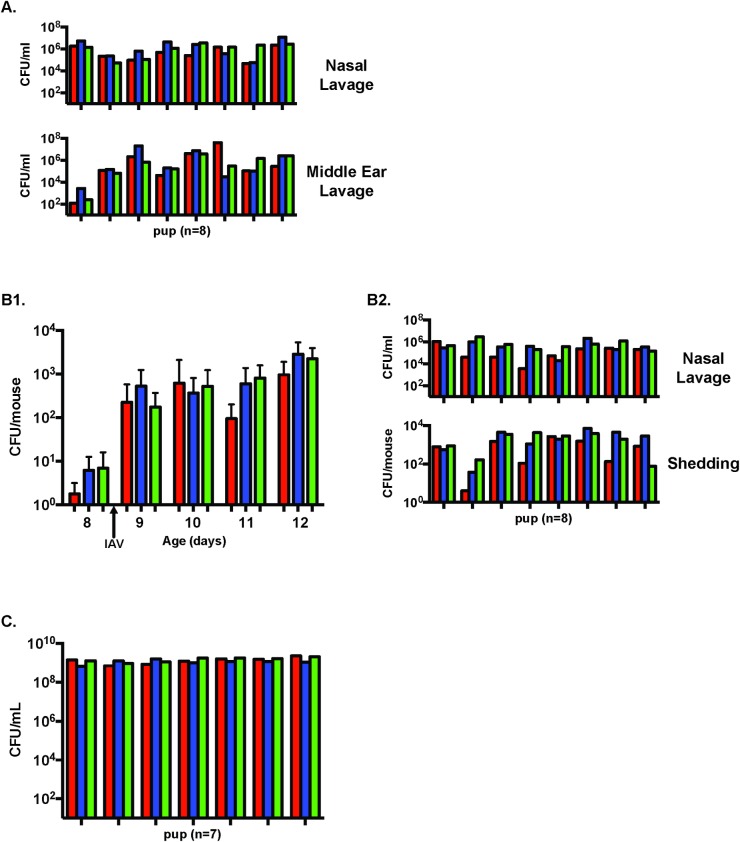
Steps in pneumococcal pathogenesis without a tight population bottleneck. See Table 1 for summary of data. **A.** Nasal colonization to the middle ear cavity from eight representative animals. Pups were infected with an equal mixture of three marked mutants on day 4 and IAV on day 8 of age. Colonization density for each mutant (shown as a different colors) was determined in nasal lavages (above) and middle ear exudate from the corresponding pup (below). Middle ear lavages of right side with each vertical tick mark on the x-axis representing one pup. **B.** In nasal secretions. Pups were infected with three mutants on day 4 and IAV on day 8 of age. B1. Daily number of shed bacteria at the age indicated for each of the three mutant strains ± S.D. B2. All three strains were detected in nasal lavages (above) and in the shedding assay in the corresponding pups (below) on day 12 of age. Each vertical tick mark on the x-axis represents one pup. **C.** In bacteremia after IP challenge. The nasopharynx was bypassed and at age day 13 pups were challenged IP with an equal inoculum of all three mutants. After 14 hours, all pups showed signs of sepsis and blood was obtained for quantitative culture.

At age 4 days, the pups were inoculated with an equal mixture of the three strains. Although there was some variation in the colonization density of each strain for individual pups, at age 12 days no single strain consistently prevailed over the others and all pups were colonized with all three strains with (Fig 2A and 2B2, upper panels) or without IAV co-infection (S2B Fig). In pilot experiments, we also established that all pups were colonized at similar levels with these strains at earlier time points. 13 of 15 middle ear samples with pneumococci following IAV co-infection also contained all three mutants (representative data from the right middle ear in Fig 2A, lower panel). There were too few mice with otitis media in the absence of IAV to assess the bottleneck size in this experimental condition. Our findings indicated that with this low inoculum and at this time point there is not a tight population bottleneck during colonization of infant mice (with or without IAV co-infection) or in the transition of bacteria to a normally sterile site (middle ear cavity) in the upper respiratory tract in the setting of IAV (summarized in **Table 1**).

**Table 1:** Conditions without a tight population bottleneck.

		Number of strains detected		
Condition (challenge route)	Agent (day inoculated)	1	2	3
Colonization (IN)	*Sp*(4) + IAV(8)	0	0	16
	*Sp*(4) + PBS(8)	0	0	18
Otitis media (IN)	*Sp*(4) + IAV(8)	0	2	13
Shedding (IN)	*Sp*(4) + IAV(8)	0	0	8
Bacteremia (IP)	*Sp*(13)	0	0	7

Data represents the number of mice in which P2396, P2397 or P2405 was detected after challenge with an equal mixture of the three isogenic mutants. *Sp*, *Streptococcus pneumoniae*. IAV, influenza A virus.

### Population bottlenecks in bacteremic infection following colonization

**Fig 3:**
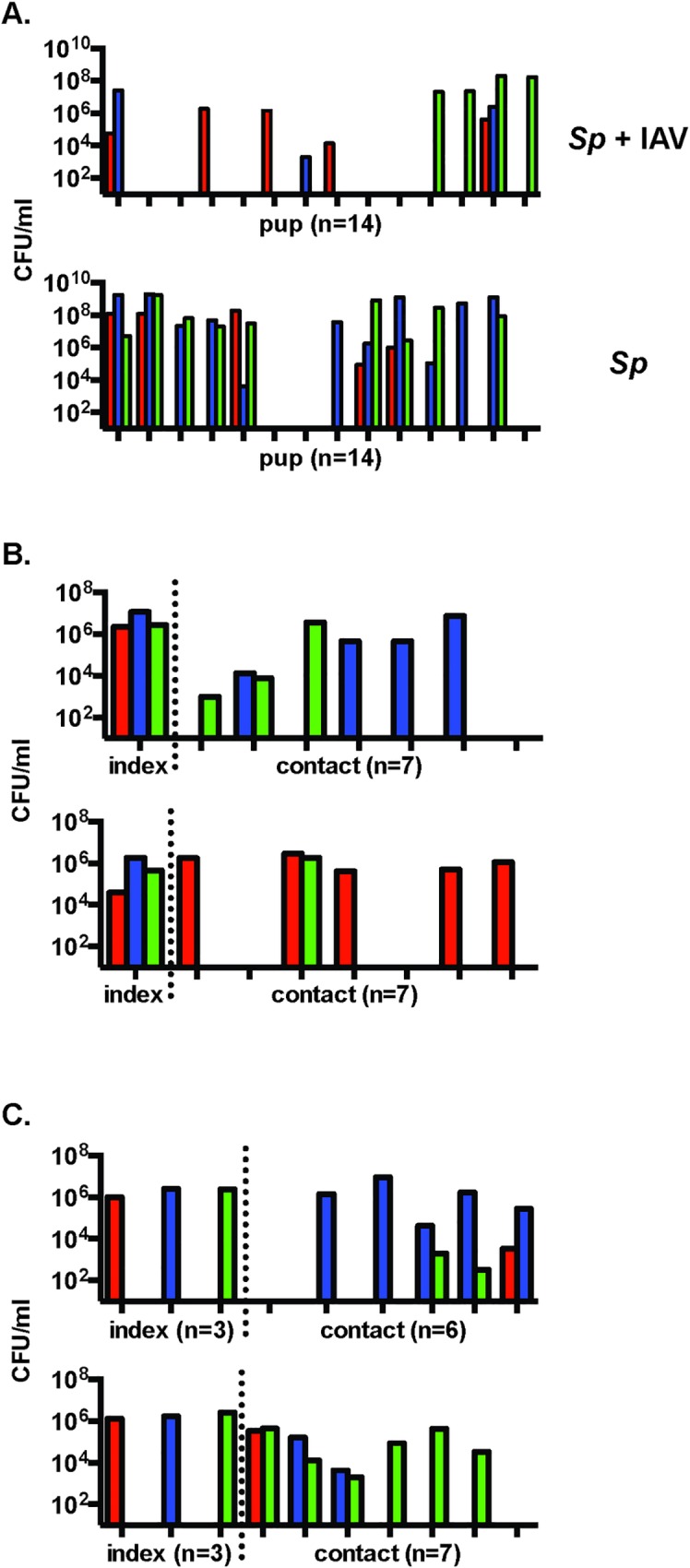
Analysis of tight population bottlenecks. **A.** A tight population bottleneck exists in bacteremia after IN challenge. Pups were inoculated with three mutant mixture on day 4 and IAV (above) or PBS (below) on day 8 of age. Data with IAV is from three representative experiments. Each vertical tick mark on the x-axis represents results of blood cultures obtained when the pups showed signs of sepsis from one pup. **B.** Most transmission events originate from a single organism. Two representative litters out of five are shown. One pup was inoculated with each of three marked mutants on day 4 of age (index mice) and returned to the littermates (contacts). **C.** Multiple entry events may occur during transmission. Three pups were each inoculated with one of the three marked mutants on day 4 of age (index mice) and returned to the littermates (contacts). **B, C.** On day 8, all pups were infected with IAV. The bacterial density in the nasal lavage of each mouse on day of age 12 is shown. Each vertical tick mark on the x-axis represents results of cultures nasal lavages from a single pup.

While all pups had three strains in nasal lavages when sacrificed, following IN challenge fewer than three mutants were detected in the majority of blood cultures with (Fig 3A, upper panel) or without (Fig 3A, lower panel) IAV co-infection. The most probable size of the bottleneck was evaluated by a mathematical model based on a Poisson distribution where the predicted and observed number of strains/mouse was compared for population bottlenecks of 1,2,3 or ≥4 cells (see Materials and Methods). This calculation suggested at some point prior to the development of bacteremia there was a population bottleneck in the setting of IAV that most often allowed passage of only a single bacterium (*w* = 1) (**Table 2**). A caveat of this observation is that this single event could represent more than one cell since the pneumococcus is a chain-forming organism. In the absence of IAV co-infection, more than a single mutant was usually detected in the bloodstream, suggesting a significantly looser bottleneck effect (*w* = 2 or 3) compared to the IAV group. The difference in the proportion of bacteremic events from a single strain with and without IAV co-infection was significant (*p* = 0.018, two-tailed Fisher’s exact test).

**Table 2:** Conditions with a tight population bottleneck.

Condition			Number of strains detected			
			1	2	3	
Bacteremia						
*Sp* + PBS	Observed		2	4	5	
	Estimated					*p*
		*w* = 1	6.01	3.59	0.407	<0.001
		***w* = 2**	**1.68**	**5.39**	**2.94**	**0.39**
		***w* = 3**	**0.528**	**4.13**	**5.35**	**0.13**
		*w*≥4	0.173	2.85	6.98	<0.001
*Sp* + IAV	Observed		12	4	1	
	Estimated					*p*
		***w* = 1**	**11.1**	**4.78**	**0.438**	**0.63**
		*w* = 2	3.24	9.27	3.77	<0.001
		*w* = 3	1.03	7.56	7.69	<0.001
		*w*≥4	0.340	5.38	10.6	<0.001
Transmission						
*Sp* + IAV	Observed		19	8	0	
	Estimated					*p*
		***w* = 1**	**14.5**	**8.90**	**1.02**	**0.28**
		*w* = 2	4.03	13.1	7.30	<0.001
		*w* = 3	1.27	9.96	13.2	<0.001
		*w*≥4	0.414	6.86	17.1	<0.001

For bacteremia, data shows the number of mice in which P2396, P2397 or P2405 was detected in blood cultures after intranasal challenge with an equal mixture of the three isogenic mutants. For transmission experiments, one index mouse/litter was challenged with the three strain mixture and data shows the number of mice in which P2396, P2397 or P2405 was detected in the contact pups. *Sp*, *S*. *pneumoniae* inoculated at age 4 days. IAV, influenza A virus inoculated at age 8 days. *w* indicates the estimated founding number of bacteria which established the population in the blood following colonization or in the nasopharynx of contact pups following transmission. Total number of pups for detection of bacteremia were n = 14 (*Sp*+PBS), n = 25 (*Sp*+IAV) and contacts for transmission n = 34 (*Sp*+IAV tested in 5 cages containing 6–7 contact pups in each cage). The estimated number of pups bacteremic or contact pups colonized with one, two or three strains was calculated by a statistical model for each number of *w* (1–4). The observed number and the estimated number were then compared by Chi-square goodness-of-fit test and the *w* with the largest *p* value (shown in bold) denotes the most probable size of the population bottleneck.

Next, the mechanisms contributing to the population bottleneck in the development of bacteremia were considered. To determine whether the bottleneck in events leading to bacteremia occurred following passage into or within the bloodstream, nasopharyngeal colonization was bypassed by systemically challenging pups with a low dose of the three strains. Since pups were too small for intravenous inoculation, this was carried out by intraperitoneal inoculation (IP), which leads to rapid bacterial uptake into the circulation. At 14 hours following IP challenge all mice were bacteremic at high levels with all three strains (**Table 1**and **Fig 2C**). Thus, sepsis appeared to develop rapidly with bacterial invasion even through it was not observed before age 12 days following IN challenge. This suggests that the bottleneck seen in bacteremia following IN challenge is likely due to events following colonization and prior to accessing the bloodstream. The need to pass through this tight bottleneck could have contributed to the long incubation period for the development of bacteremia following IN challenge. When IN bacterial challenge was shifted from age 4 to 8 days, sepsis was still observed beginning at age 13 days, suggesting an age-dependent susceptibility to invasive infection following colonization (**Fig 1D**).

Because seeding of the bloodstream could occur following invasion from either the upper or lower respiratory tract, we determined whether this delay in the development of bacteremia was due to a requirement for infection of the lungs following colonization. However, regardless of IAV co-infection, when sampled at age 12 days just prior to the onset of bacteremia, detection of pneumonia by culture of lung homogenates was unusual (1 out of 10 mice).

The tight population bottleneck in the setting of IAV could be due to the selection of a subpopulation of pneumococci because of a requirement for *de novo* genetic adaptation. Alternatively, passage through a bottleneck could require selection among pre-exiting variants. To examine these possibilities, we examined whether a strain that had passed through the bottleneck showed increased fitness for bacteremic infection during rechallenge. Single strain blood isolates were mixed with strains containing the other two resistance markers and re-inoculated IN into pups at age 4 days. However, we found that a strain that had previously passed through the bottleneck was no more likely to cause bacteremia upon rechallenge compared to strains that had not passed through the bottleneck (representative data in S3 Fig upper panel).

It was concluded that invasive infection following colonization requires passage through a population bottleneck whose size is determined primarily by host factors and more restricted in the setting of IAV co-infection.

### Population bottlenecks in host-to-host transmission

To evaluate whether a bottleneck occurs during a transmission event, the index pup was infected IN with an equal mixture of the three strains and returned to the litter. Since there was no detectable transmission among the PBS control group (**Fig 1E**), only IAV co-infection was tested. Two representative litters out of five are shown in **Fig 3B**. Although all three strains colonized the nasal cavity of the index pup similarly, only a single strain established colonization in most (19 of 27 acquiring the organism) contact mice, suggesting a single bacterium or bacterial chain (*w* = 1) was likely responsible for the transmission event (**Table 2**). The outcome of transmission from a single strain was significantly more likely than other outcomes (*p* = 0.006, two-tailed Fisher’s exact test). There was no consistent correlation between the strain colonizing the index pup at the highest density and the strain(s) detected in contact pups, suggesting that transmission is a stochastic event.

To define the step in transmission with a tight population bottleneck, we analyzed exit from the donor host, entry into the recipient host, and establishment in the new host. In IAV co-infected mice, there was daily shedding of each of the three mutants (**Fig 2B1**) and all pups were colonized by and shed all three strains (**Fig 2B2**), indicating a lack of a bottleneck during the exit of bacteria **(Table 1**). To examine whether there is a bottleneck during entry into the recipient, the experimental conditions were changed. Three index mice, each colonized at age 4 days with one of the three mutants, were used per litter to test whether there could be multiple entry events in the same recipient. Although there was still a tight bottleneck in transmission (*w* = 1), 6 of 11 contact pups acquiring the organism became colonized with more than one strain (**Fig 3C** and **Table 3**). This demonstrates that multiple entry events may occur and made it unlikely that strains compete at the initial entry step. In contrast to the contact mice, none of the singly colonized index mice acquired another strain. This finding suggests that once high density colonization is established further acquisition events are excluded. To confirm this observation, the experimental conditions were changed again. All the pups in a litter were colonized with a single strain–one third with each of the three mutants. No acquisition events (0 out of 17) between pups with established colonization were detected. This finding correlated with an increase in the bacterial inoculum required for establishing colonization in the majority of previously colonized compared to naive pups (>1000 CFU vs. <10 CFU). It was concluded that the tight bottleneck in transmission occurs following exit and before establishment suggesting that on average only a single shed organism/chain prevails in entering the recipient and founding a population that once established effectively outcompetes newcomers.

**Table 3:** Population bottleneck in transmission: Effects of the number and genotype of index mice.

Condition			Number of strains detected			
			1	2	3	
Three wildtype index mice (one mutant/index)						
contact	Observed		5	6	0	
	Estimated					*p*
		***w* = 1**	**5.15**	**3.79**	**0.49**	**0.41**
		*w* = 2	1.39	4.87	3.18	<0.01
		*w* = 3	0.43	3.56	5.44	<0.001
		*w*≥4	0.14	2.40	6.89	<0.001
One *tlr2* ^*-/-*^ index mouse (three mutants/ index)						
wildtype contact	Observed		5	5	1	
	Estimated					*p*
		*w* = 1	8.23	2.42	0.17	<0.05
		***w* = 2**	**2.50**	**6.47**	**1.84**	**0.20**
		*w* = 3	0.81	5.58	4.43	<0.001
		*w*≥4	0.27	4.08	6.74	<0.001
*tlr2* ^*-/-*^ contact	Observed		5	6	2	
	Estimated					*p*
		*w* = 1	7.41	4.14	0.45	<0.05
		***w* = 2**	**2.09**	**6.54**	**3.36**	**0.41**
		*w* = 3	0.66	5.08	6.25	<0.001
		*w*≥4	0.22	3.53	8.24	<0.001

Data shows the number of contact mice in which P2396, P2397 or P2405 was detected after intranasal challenge of index mice. The genotype of the index and contact mice is indicated (wildtype BL6 or *tlr2* ^*-/-*^ *)*. Two models were tested: either three pups (index mice) were each inoculated with one of the three marked mutants and returned to the littermates (contacts) or one pup (index mouse) was inoculated with an equal mixture of the three isogenic mutants and returned to the littermates (contacts). Index mice were given *Sp*, *S*. *pneumoniae* inoculated at age 4 days. IAV, influenza A virus was given at age 8 days to all pups. *w* indicates the estimated founding number of bacteria that established the population in the nasopharynx of contact pups. Total number of contact mice with three wildtype index mice, *tlr2* ^*-/-*^ index/wildtype contacts and *tlr2* ^*-/-*^ index/*tlr2* ^*-/-*^ contacts were n = 13, n = 20 and n = 17 respectively. The estimated number of contact pups (wildtype BL6 or *tlr2* ^*-/-*^ *)* colonized with one, two or three strains was calculated by the statistical model for each number of *w* (1–4). The observed number and the estimated number were then compared by Chi-square goodness-of-fit test and the *w* with the largest *p* value (shown in bold) denotes the most probable size of the population bottleneck.

### Factors contributing to the tight bottleneck in transmission

A strain that successfully passed through the bottleneck in transmission, was no more likely to be transmitted following IN rechallenge (representative data in S3 Fig lower panel). It was unlikely, therefore, that *de novo* genetic adaptation or selection among pre-exiting stable variants was a requirement for passage through the tight bottleneck in transmission.

**Fig 4:**
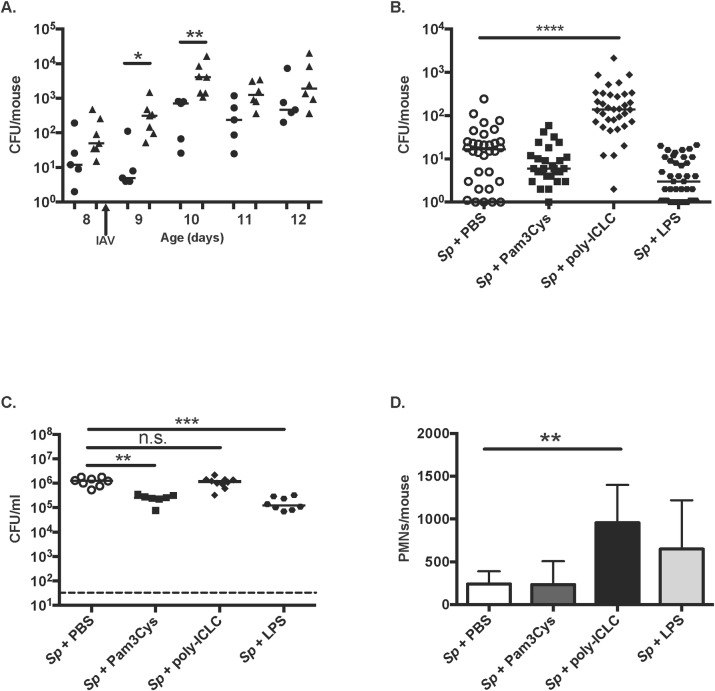
Increased pneumococcal shedding among *tlr2* ^*-/-*^ mice or by daily treatment with Toll-like receptor (TLR) agonists. **A.** Wildtype (black circle) or *tlr2* ^*-/-*^ pups (black triangle) were infected with strain P1547 on day 4, and IAV on day 8 of age as indicated. Daily nasal secretions were collected between day 8–12 of age. Each dot represents a single mouse. Statistical differences between the two groups in each day were evaluated by using Mann-Whitney U test. * *p*<0.05 and **p<0.01. **B, C, D.** Wildtype pups were infected with strain P1547 on day 4, and given a daily IN dose of PBS, Pam3Cys, poly-ICLC or LPS between days 8 and 12. Statistical analyses between four groups were performed by Kruskal-Wallis test (Dunn’s multiple comparison test). **p<0.01, *** *p*<0.001, **** *p*<0.0001 and n.s. not significant. **B.** Shedding (combined values from days 9 through12) was compared by quantitative culture of secretions. **C.** Colonization was compared by quantitative culture of nasal lavages obtained at day 12 of age. **D.** The number of neutrophils (PMNs) in the nasal lavages were counted by flow cytometry by gating on CD11b^+^, Ly6G^+^ events.

To test the role of inflammation in the bottleneck in transmission, experiments were repeated in IAV co-infected *tlr2* ^*-/-*^ mice, which we previously showed shed and transmit more readily due to increased inflammation in response to IAV [14]. One *tlr2* ^*-/-*^ pup/litter was infected with the three strain mixture and co-housed with wildtype contacts. Colonization was equivalent to levels in wildtype index mice and again similar for each of the three mutants (S4A and S4B Fig). Multiple strains were detected in the nasopharynx of the majority of the wildtype contacts acquiring the organism (**Table 3**). A similar result was obtained when an index *tlr2* ^*-/-*^ pup was co-housed with *tlr2* ^*-/-*^ contacts. IAV infected *tlr2* ^*-/-*^ index mice shed at significantly higher levels compared to wildtype controls on days of age 9 and 10 (**Fig 4A**), providing an explanation for increased numbers of bacteria reaching the contacts. The size of bottleneck for *tlr2* ^*-/-*^ index mice was estimated to be at least *w* = 2 regardless of the genotype of the contacts and was less tight compared to wildtype index mice (**see Table 2**). The proportion of colonized contact mice with a single mutant was greater for experiments with wildtype compared to *tlr2* ^*-/-*^ index mice (two-tailed Fisher’s exact test, *p* = 0.05). The looser bottleneck in *tlr2* ^*-/-*^ mice with an altered response to IAV, demonstrated that the characteristics of the host response are a determining factor in the size of the bottleneck in transmission. The effect of IAV on shedding was partially recapitulated by daily IN dosing with the prototypical viral TLR3 agonist poly-ICLC (**Fig 4B**). In contrast, the prototypical bacterial agonists for TLR2 (Pam3Cys) and TLR4 (LPS) failed to stimulate shedding. Bacterial shedding sufficient to promote transmission requires the effect of inflammation on secretions and a high density of colonization [14]. Among the TLR agonists tested only poly-ICLC was able to stimulate inflammation as determined by the magnitude of the neutrophil response without negatively impacting colonization density (**Fig 4C and 4D**). This provided further evidence that specific innate immune responses to IAV dictate key determinants of the bottleneck in transmission.

## Discussion

This report describes an animal model that allows for the study of many of the consequences of bacterial colonization including localized and systemic disease and host-to-host transmission. We utilized this model to examine the effects of a clinically important viral co-infection. We found that IAV increases the burden of colonizing pneumococci consistent with prior observations in infant mice [18]. A similar effect had been shown in adult mice and was attributed to the increased availability of the nutrient sialic acid in secretions stimulated by IAV [16]. The greatly increased bacterial shedding and transmission in the setting of IAV described in the current report was out of proportion to the relatively modest difference in colonization density and could be attributable to the effect of the virus on secretions enhancing bacterial egress from the host. The higher rate of otitis media in the setting of IAV is consistent with prior reports and could be due to either an increased number of colonizing organisms or eustachian tube dysfunction [19][20].

The use of the infant mice avoided the ‘limited effective population’ that has been proposed in colonization of adult mice and that could hamper the analysis of population bottlenecks [21]. In this regard, we established that following a low inoculum dose all infant mice were colonized by all three of the strains and that each of three strains was shed every day post-challenge. This enabled a comprehensive analysis of bottlenecks for events following colonization. A limitation of our study using infant mice was the inability to obtain multiple samples at different times during infection from the same animal. Thus, we could only assess population bottlenecks at a single time point. Our study suggests that the host response rather than microbial factors are the main determinants of the size of these population bottlenecks.

In the case of bacteremia, the population bottleneck in passage from the mucosal surface to the bloodstream was tight, but even more restricted in the setting of IAV co-infection. This increased barrier could be due to the mucosal inflammatory response that accompanies IAV infection. The effect on IAV on restricting the size of the bottleneck in bacteremia added further support that this bottleneck exists at the mucosal interface since IAV infection does not cause viremia. It is interesting that the onset of sepsis/bacteremia was delayed up to eight days after colonization was established and did not occur until about twelve days age regardless of the duration of colonization. This age-related invasive infection in infant mice differs from congenic adult mice, which become septic within 2–4 days following IN challenge with the same pneumococcal isolate [22]. The overall inflammatory milieu of the respiratory mucosa is elevated in infant compared to young adult mice [16]. This more inflamed state could tighten the bottleneck and result in a more delayed onset of bacterial invasion in infants. An alternative explanation is that additional events are required during infant infection and raises the possibility of a reservoir for the organism outside the upper respiratory tract from which seeding of the bloodstream occurs. This reservoir did not appear to reside in the lower respiratory tract. A reservoir was also proposed by Moxon and Murphy, who demonstrated single cell bottlenecks in bacteremic infection of infant rats nasally challenged with *Haemophilus influenzae* type b following an eclipse phase [23]. We previously described how TLR- or TGFβ-signaling in polarized epithelial cells and in vivo opens tight junctions between cells and facilitates bacterial invasion [24]. It would be interesting to test whether such signaling has the effect of loosening the size of the bottleneck in bacteremia. Lastly, our data showing the lack of tight bottleneck following IP challenge with a low inoculum contrasts with those of Gerlini et al, who described a tight bottleneck in pneumococcal bacteremia of adult mice following intravenous challenge with a high inoculum (3×10^5^ CFU) with a different bacterial strain and in a different mouse line [25]. Of note, their challenge with ~10^3^ more pneumococci appears to overwhelm splenic clearance. The tight bottleneck we observed following invasion from the mucosal surface, where pneumococcal bacteremia originates, suggests that a large bolus of pneumococci may not access the bloodstream in this manner. Also, the Gerlini et al study followed bacteremia with multiple blood cultures over 3 days; whereas our use of infant mice precluded multiple sampling and all pups had to be sacrificed within hours of acquiring invasive infection due to the rapid onset of sepsis.

In the case of transmission, the identification of a tight bottleneck explains the requirement for IAV co-infection. This was attributed to mucosal inflammation and increased secretions in the setting of IAV facilitating bacterial exit and transit to a new host. The use of mice lacking TLR2-signaling needed to control responses to IAV that showed increased bacterial shedding and a looser bottleneck confirmed these effects of viral infection [14][26]. This correlation between the number of shed bacteria and the incidence of transmission provides an explanation why IAV co-infection is required to raise transmission events to a detectable level. The effect of IAV on nasal secretions could be explained by its stimulation of TLR3-signaling since IN administration of the analog of viral dsRNA, poly-ICLC, was sufficient to increase bacterial shedding in the absence of viral infection. Other pathways involved in innate responses to IAV may also contribute to these effects on the host. In contrast, intranasal administration of bacterial PAMPs were ineffective in stimulating an inflammatory response sufficient to enhance bacterial shedding. These findings suggest there may be specificity for signaling pathways triggered by viral infection. We propose that in this model without IAV the number of shed bacteria would be generally insufficient to surpass the threshold required to transit the tight bottleneck. A further consideration is that the higher inoculum required for establishing colonization in adult mice is unlikely to pass through this tight bottleneck explaining the lack of transmission beyond the infant period [16]. The demonstration of a single cell bottleneck in transmission provides context for understanding some its requirements. There did not appear to be a requirement for multiple organisms to enter a new host or for genetic adaptation to establish colonization upon entry into a new host. Rather our findings suggest that events prior to entry are critical. In particular, since transmission rates correlate with the density of shed organisms, it appears that there must be a sufficient number of bacteria exiting their niche on the mucosal surface for a single organism/chain to succeed in reaching the nasal mucosa of a new, susceptible host. This level of shedding might have to be actively induced by environmental factors in the host as demonstrated by the requirement for IAV co-infection in this model system. Shed organisms must also survive for a sufficient period to encounter a new host. Bacterial constituents that allow for survival between hosts are poorly understood, although a prior report documented that this species is highly resistant to desiccation [27]. The small number of pneumococci able to succeed in being transmitted is likely why additional transmission events were not observed in pups where colonization was already established, since the minimum colonizing dose is greater than two-logs higher in this setting. A tight bottleneck in transmission also explains why close contact between individuals, and in particular with young children, is typically required for pneumococcal ‘contagion’. We are currently determining whether there are specific bacterial factors that actively induce shedding and enhance transmission.

The plasticity of the pneumococcus is the major challenge to prevention of disease through immunization and control by antibiotics. Here we demonstrate two key steps in its pathogenesis, transmission and bacteremia following colonization, where its population size is highly restricted and, therefore, its ability to take advantage of its marked genetic diversity and adapt by genetic exchange is minimized.

## Materials and Methods

### Ethics statement

This study was conducted according to the guidelines outlined by National Science Foundation Animal Welfare Requirements and the Public Health Service Policy on the Humane Care and Use of Laboratory Animals. The Institutional Animal Care and Use Committee (IACUC) at New York University approved these animal studies. New York University's IACUC oversees the welfare, well-being and proper care and use of all vertebrate animals used for research and educational purposes at NYU Langone Medical Center and School of Medicine. New York University's IACUC Assurance Number is A3435-01. The approved protocol numbers for this project are 150216–01 and 150520–01.

### Bacterial strain construction and growth conditions

P1547, a serotype 6A clinical isolate that causes sepsis in adult mice following intranasal challenge, was used as a wildtype strain [22]. Strains P2396, P2397 and P2405, respectively, were constructed by transforming P1547 with plasmid DNA encoding an immunoglobulin A1 (IgA1) protease interrupted with an antibiotic resistant cassette to erythromycin (*rRNA adenine N-6-methyltransferase*), spectinomycin (*spectinomycin adenyltransferase*) or kanamycin (*3'*, *5'-aminoglycoside phosphotransferase type III*). We confirmed the insertion of the antibiotic cassettes in IgA1 protease gene by sequencing using primers (for P2396 and P2397; 5’-TCAGTAGGACTTGTATCTGC-3’ and 5’-TGGATTTAGCAATAGACGC-3’, for P2405; 5’-CCTGTCAGATCATCTCATCG-3’ and 5’-CCATTGAATAGTAGCCATTG-3’). Mutants were selected on 5% sheep blood agar plates containing erythromycin (0.1μg/ml), spectinomycin (200μg/ml) or kanamycin (500μg/ml).

Pneumococcal strains were grown statically in Tryptic Soy (TS) broth (BD, Franklin Lakes, NJ) to mid-exponential phase at 37°C. When the bacterial culture reached the desired optical density at 620nm, cells were washed and diluted in sterile PBS for inoculation. Quantitative culture was performed by plating 10-fold serial dilutions in triplicate on selective plates. Plates were incubated overnight at 37°C with 5% CO_2_. Bacteria were stored in 20% glycerol at -80°C.

### Mice

All mice were bred in our facility and maintained as a dam with pups from the same litter (average size 6–8). Wildtype C57BL/6 mice and *tlr2* ^*-/-*^ mice were originally obtained from The Jackson Laboratory (Bar Harbor, ME) and bred and maintained in a conventional animal facility. Polyinosinic-polycytidylic acid condensed with poly-l-lysine (poly-ICLC, Hiltonol), a synthetic analog of viral dsRNA, was supplied by Oncovir Inc. Pam3CysSerLys4 (Pam3Cys), a synthetic triacylated lipoprotein (Invivogen) and lipopolysaccharides (LPS) from *Salmonella enterica* (Sigma-Aldrich) were resuspended in endotoxin free water to a concentration of 5mg/ml. Where indicated, mice were given a daily IN dose of poly-ICLC (2.0 μg/pup), Pam3Cys (10 μg/pup) or LPS (10 μg/pup) or vehicle control from age 8 to 12 days.

### Colonization of infant mice

Four day old pups were inoculated IN with a total of ~9,000 CFU of *S*.*pneumoniae* suspended in 3μl of PBS using a blunt pipette without anesthesia. On day eight of age, pups were inoculated IN either with influenza A virus/HKx31 strain (H3N2) (2×10^2^–2×10^4^ TCID_50_) suspended in 3μl of PBS or an equal volume of PBS. Influenza A virus was grown in the allantoic fluid of 10-day embryonated chicken eggs (B&E Eggs) and stored at -80°C. Viral concentrations for infection were determined by titration in Madin-Darby Canine Kidney cells, as described previously [28].

At age 12 days, pups were euthanized by CO_2_ asphyxiation, the upper respiratory lavaged with 200μl of sterile PBS from a needle inserted in the trachea, and fluid collected from the nares. To detect P1547 or its derivatives in lavages, aliquots were plated on 5% sheep blood agar containing neomycin (20μg/ml) or the selective antibiotics listed above to minimize contamination. The limit of detection was 33 CFU/ml unless otherwise noted.

The colonizing dose for strain P2405 was determined using ten-fold serial dilutions in PBS inoculated IN to pups (≥3/dose) given at age 8 days with colonization quantified in lavages at age 12 days. Where specified mice were either sham (PBS) inoculated or given a colonizing dose of strain P2397 at age 4 days to test the effect of pre-existing colonization.

### Detection of otitis media

Experiments to detect otitis media were carried out with the same infection schedule as described above. On day 12 of age, pups were euthanized to collect nasal lavages and middle ear lavages. Using a dissecting microscope, both middle ear cavities were washed with 3μl of PBS with a sharp sterile tip after myringotomy. The detection limit for quantitative culture for each sample was 3333 CFU/ml.

### Detection of pneumonia

Experiments to detect pneumonia were carried out with the same infection schedule as described above. On day 12 of age, whole lung tissue, nasal lavages and blood were collected in order to minimize contamination between sites. The lung tissue was homogenized in 1ml of sterile PBS and aliquots serially diluted for quantitative culture.

### Detection of bacteremia

Bacteremia was assessed following infection by two different routes. For the intranasal challenge route, four-day old pups were infected IN with *S*.*pneumoniae*, followed by either IAV or PBS on day eight of age. The pups were then observed every 12 hours after IAV or PBS inoculation for signs of sepsis (decreased motor activity, shivering or weight loss). Ill-appearing pups were immediately euthanized to obtain nasal lavages and collect blood samples via cardiac puncture for cultured (50 μl volume). The limit of detection to quantify bacteremia was 33 CFU/ml of blood. Pups without signs of sepsis were euthanized on 18 day of age and nasal lavage and blood cultures obtained. Bacteremia was assessed only at the time of sacrifice because the small size of pups precluded repeated sampling of blood for culture.

For the IP challenge route, 13 day old pups were injected with ~450 CFU of *S*.*pneumoniae* (~150 CFU of each mutant) suspended in 50μl of sterile PBS. At 14 hours post-infection, pups were euthanized and blood samples were collected by cardiac puncture for quantitative culture.

### Transmission in infant mice

The experimental schedule was based on a previously described study [14]. The schedule was modified and transmission was assessed at age 12 rather than 14 days to minimize the possibility of transmission from a newly colonized contact mouse to another contact mouse. Except where indicated, one pup in each litter was randomly selected as the index mouse and at age 4 days infected IN as described above. The index mouse was then returned to the litter and housed with the dam and other uninfected pups (contact mice). When the litter was 8 days of age, all pups were inoculated IN either with IAV or PBS. In co-infection studies, all pups within a litter were given IAV to eliminate the potentially confounding effect of viral transmission among pups. To detect bacterial transmission from the index to contact pups, all pups were euthanized at age 12 days and nasal lavages were collected and plated on the selective medium.

### Quantification of bacterial shedding

The infection schedule was the same as described above for colonization experiments. From day 8–12 of age, nasal secretions were cultured in samples collected by gently tapping (10 times) the nares onto TS agar containing neomycin (20 μg/ml) and catalase (6,300U/plate) (Worthington Biochemical Corporation, NJ) [14]. The sample was then evenly spread across the plate using a sterile swab for quantitative culture. When a mixture of marked strains was used, the colonies on TS neomycin plate were individually patched on selective agar plates to determine the ratio of each mutant.

### Flow cytometry

For evaluating an inflammatory status in the nasal cavity, nasal lavages were stained with the following antibodies: anti-CD11b-V450 (BD), anti-Ly6G-PerCP (BD) and anti-CD45-APC Cy7 (BD) after FcR blocking with anti-CD16/32 (BioLegend). Cells were then fixed with 4% paraformaldehyde until analysis. BD LSR II was used for flow cytometry and analyzed with FlowJo software.

### Statistical analyses

Mann-Whitney U test was used for comparisons between two groups. Kruskal-Wallis test was used for comparisons between three or more groups. The time course of sepsis was compared using the Kaplan-Meier’s log-rank test. All statistical analyses were performed using Graph Pad Prism 6 software.

For calculating the size of the bottleneck, we utilized the mathematical model previously reported by Gerlini et al and Margolis et al. [25][29]. In brief, this model is an approximation of taking out balls from a box containing equal number of three different colored balls, which follows a Poisson distribution. Let *k* be the number of independent events, the probability that the events would occur *k* times is calculated by $P(k)=e^{-\lambda }\frac{\lambda^{k}}{k!}$. The coefficient number *λ* is calculated by *P*(0) = *e* ^−*λ*^, which is approximated to the ratio of non-affected mice. Let *w* be the number of bacteria that established a population in each infectious site or the nasopharynx of a new host, also let *i* be the number of mutants detected in the target site in each experiment. *P*(*i*|*w*,*k*) follows Bernoulli distribution which is extended probability theory of binomial distribution. By considering each case of *i* = 0, *i* = 1, *i* = 2 and *i* = 3, each probability is calculated as below.

*i* = 0; no mutants observed; *P*(*i* = 0|*w*,*k*) = 0,

*i* = 1; one mutant observed; $P(i=1|w,k)={\left(\frac{1}{3}\right)}^{wk-1}$,

*i* = 2; two mutants observed; $P(i=2|w,k)={\left(\frac{1}{3}\right)}^{wk-1}(2^{wk}-2)$,

*i* = 3; three mutants observed;

P(i=3|w,k)=1−P(i=0|w,k)−P(i=1|w,k)−P(i=2|w,k)

The sum of the probability of *k* = 0,1,2,3…, *P*(*i*|*w*) is calculated as below.

$$P(i=0|w)=\sum_{k=0}^{\infty }P(k)∙P(i=0|w,k)=P(k=0),$$

$$P(i|w)=\sum_{k=0}^{\infty }P(k)∙P(i|w,k)=\sum_{k=1}^{\infty }P(k)∙P(i|w,k)\;for\;i=1,2,3$$

Because *P*(*k*) becomes negligibly smaller as *k* becomes larger, the cases of *k*>3 were excluded from the calculation as below.

$$P(i|w)≅\sum_{1}^{3}P(k)∙P(i|w,k)\;for\;i=1,2,3$$

Eventually the estimated number of *w* = 1, *w* = 2, *w* = 3… can be calculated by multiplying the number of total mice and *P*(*i*|*w*). We determined the most likely value of *w* by comparing with the estimated number and the observed number using the Chi-square goodness-of-fit test.

## Supporting Information

S1 Fig *In vitro* growth characteristics of three isogenic mutant strains. **A.** Individual cultures of three mutant strains were propagated in TS broth at 37°C and the optical density (OD_620_) measured. Values are based on five determinations ± S.D. **B.** Co-culture of three mutant strains in TS broth. Log phase bacteria were inoculated equally into fresh TS broth and grown at 37°C until reaching an OD_620_ of 0.8 and then plated on selective media. Values are based on five determinations ± S.D. n.s. not significant by one-way ANOVA test.
(TIF)

S2 Fig Co-colonization of three isogenic mutant strains. Pups were infected with an equal mixture of three marked mutants at day 4 of age. Nasal lavages were collected on day 12 of age and the density of each mutant quantified by plating on selective media. Three different mutants with antibiotics resistant marker, P2396 (erythromycin resistant), P2397 (spectinomycin resistant) and P2405 (kanamycin resistant), are depicted in red, blue and green, respectively. **A.** The density of each of the mutants in nasal lavages with median value indicated. Each symbol denotes an individual pup. n.s. not significant by one-way ANOVA. **B.** Pup by pup comparison of the colonization of each of the three mutants (n = 18 mice).
(TIF)

S3 Fig Lack of within-host adaptation after traversing tight population bottlenecks in bacteremia or transmission. ***Upper panel*.** A strain obtained (marked by an asterisk) from a pup bacteremic with a single mutant was mixed with the other two mutants and rechallenged intranasally. One of seven representative experiments is shown. All pups were colonized with an equal mixture of the three mutants and their blood cultured when septic or at the time of sacrifice. Each vertical tick mark on the x-axis represents results of cultures blood from a single pup. *Lower panel*. A strain obtained (marked by an asterisk) from a contact pup that had been infected with a single mutant was mixed with the other two mutants and rechallenged intranasally. A single index pup was colonized and the ability of the three mutants to be transmitted to contact pups compared. One of three representative experiments is shown. Each vertical tick mark on the x-axis represents results of cultures nasal lavages from a single pup.
(TIF)

S4 Fig Pneumococcal colonization among *tlr2* ^*-/-*^ mice. **Pups were infected with *S*.*pneumoniae* on day 4 and IAV on day 8 of age.** Nasal lavages were collected on day 12 of age and the density of colonized pneumococci quantified. **A.** Wildtype and *tlr2* ^*-/-*^ pups infected with P1547. n.s. not significant (Mann-Whitney U test). **B.** *tlr2* ^*-/-*^ index pups (n = 6) infected with an equal mixture of P2396, P2397 and P2405. Repeated measures (RM) one-way ANOVA test was used for statistical analysis. n.s. not significant.
(TIF)
