# Supplementary figures and images for: Single Cell Bottlenecks in the Pathogenesis of Streptococcus pneumoniae
Source: PLoS Pathog. 2016 Oct 12;12(10):e1005887. doi: 10.1371/journal.ppat.1005887 (PMC5061371; doi:10.1371/journal.ppat.1005887)

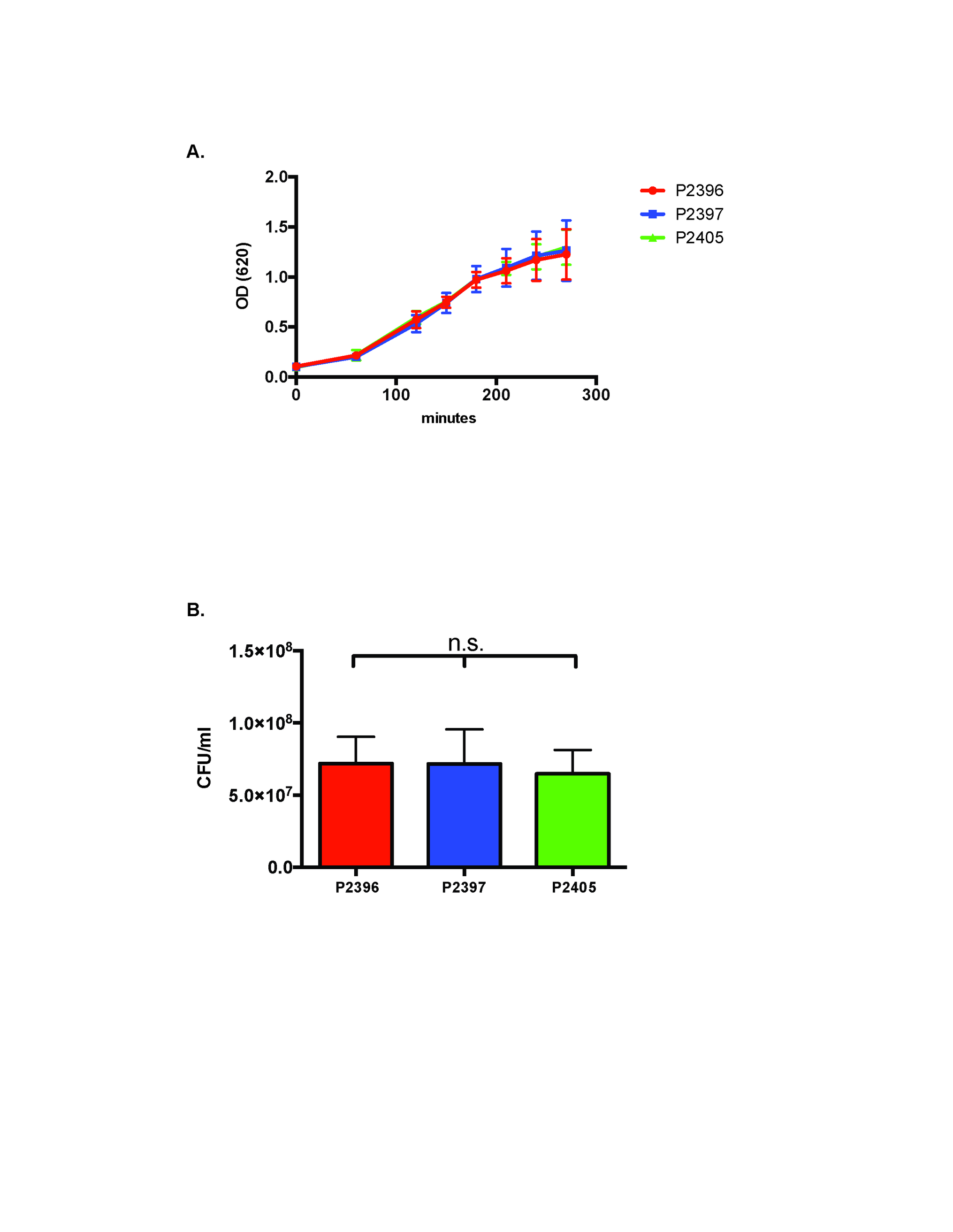

Supplement: S1 Fig — A. Individual cultures of three mutant strains were propagated in TS broth at 37°C and the optical density (OD620) measured. Values are based on five determinations ± S.D. B. Co-culture of three mutant strains in TS broth. Log phase bacteria were inoculated equally into fresh TS broth and grown at 37°C until reaching an OD620 of 0.8 and then plated on selective media. Values are based on five determinations ± S.D. n.s. not significant by one-way ANOVA test. (TIF) [file ppat.1005887.s001.tif]

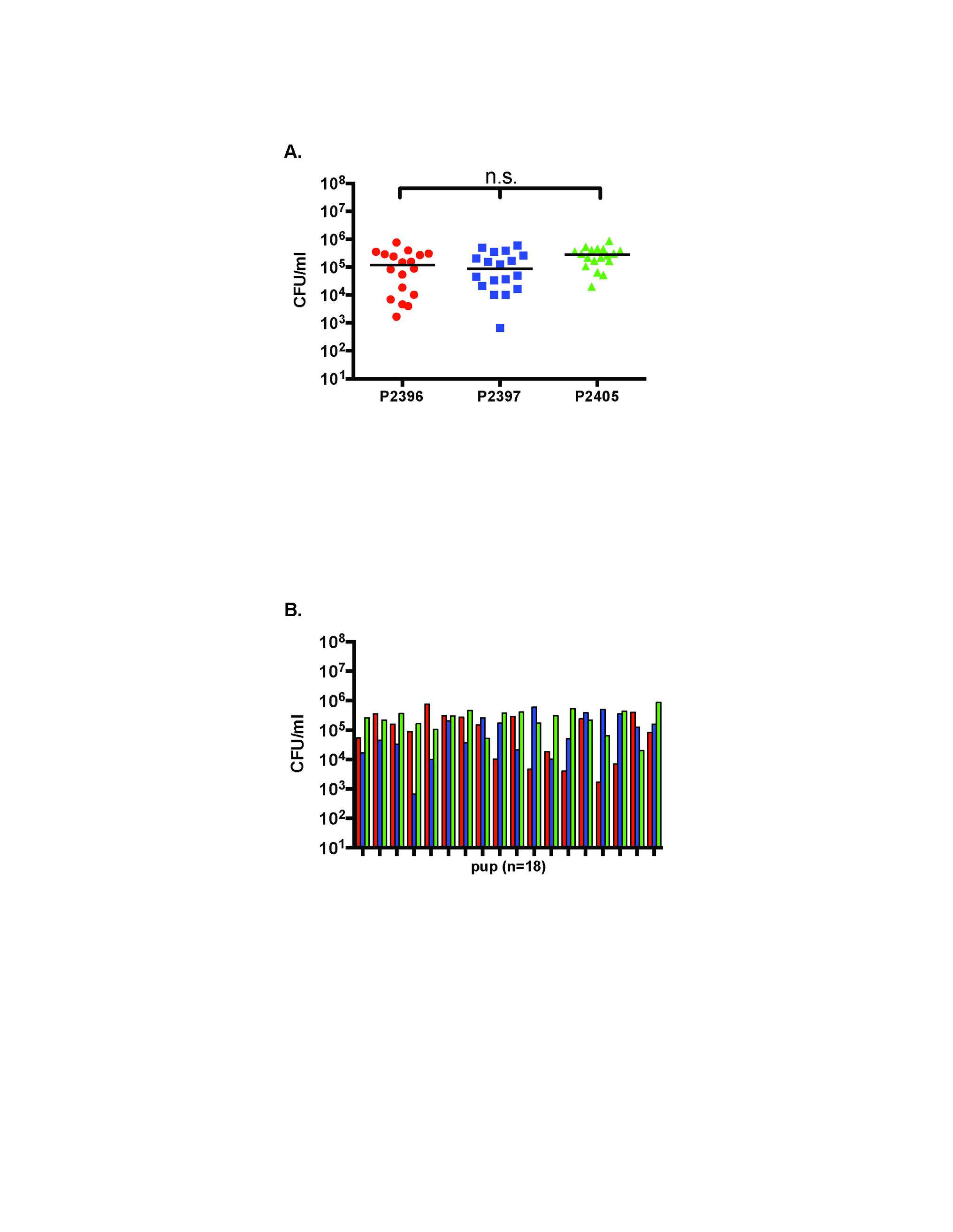

Supplement: S2 Fig — Pups were infected with an equal mixture of three marked mutants at day 4 of age. Nasal lavages were collected on day 12 of age and the density of each mutant quantified by plating on selective media. Three different mutants with antibiotics resistant marker, P2396 (erythromycin resistant), P2397 (spectinomycin resistant) and P2405 (kanamycin resistant), are depicted in red, blue and green, respectively. A. The density of each of the mutants in nasal lavages with median value indicated. Each symbol denotes an individual pup. n.s. not significant by one-way ANOVA. B. Pup by pup comparison of the colonization of each of the three mutants (n = 18 mice). (TIF) [file ppat.1005887.s002.tif]

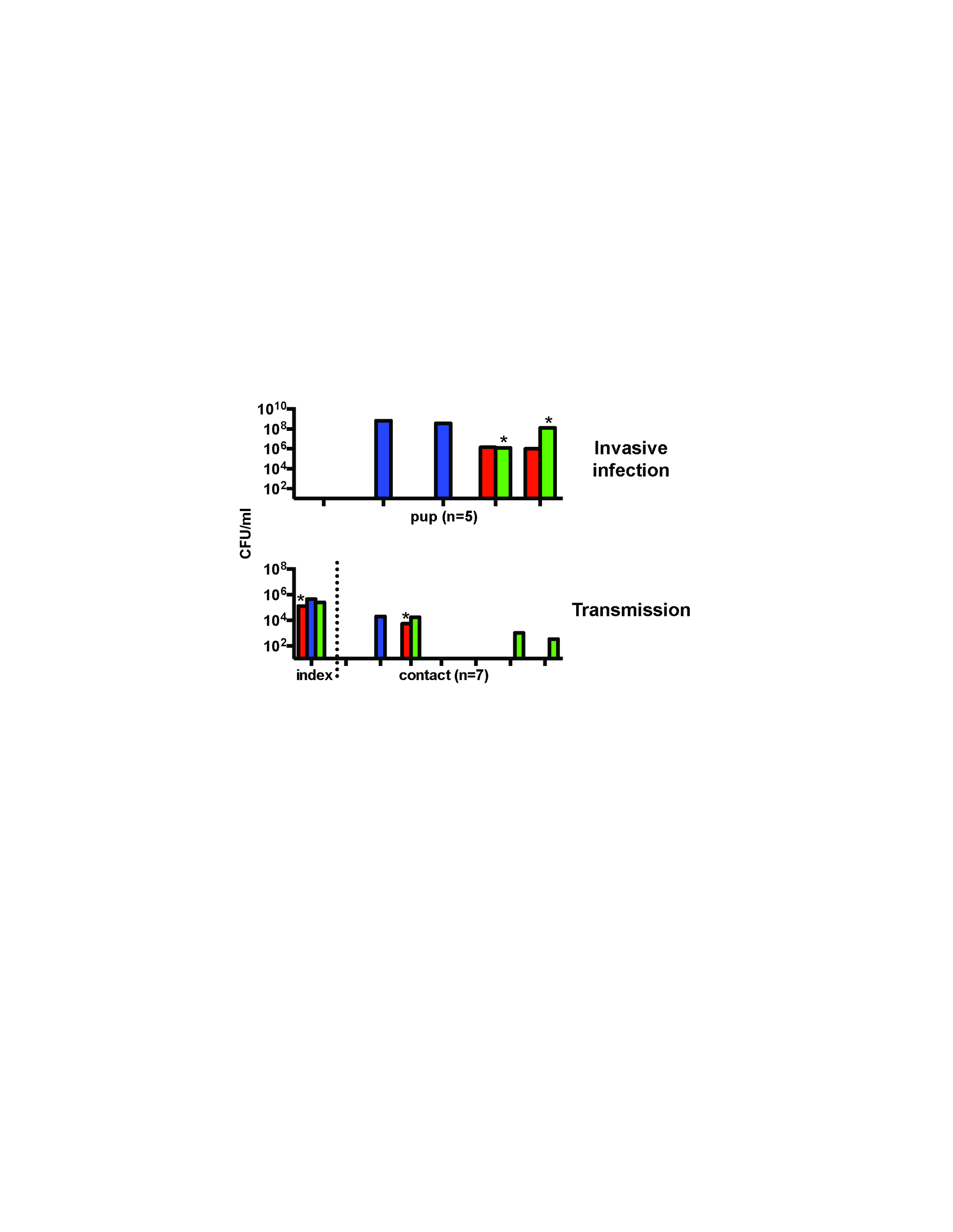

Supplement: S3 Fig — Upper panel. A strain obtained (marked by an asterisk) from a pup bacteremic with a single mutant was mixed with the other two mutants and rechallenged intranasally. One of seven representative experiments is shown. All pups were colonized with an equal mixture of the three mutants and their blood cultured when septic or at the time of sacrifice. Each vertical tick mark on the x-axis represents results of cultures blood from a single pup. Lower panel. A strain obtained (marked by an asterisk) from a contact pup that had been infected with a single mutant was mixed with the other two mutants and rechallenged intranasally. A single index pup was colonized and the ability of the three mutants to be transmitted to contact pups compared. One of three representative experiments is shown. Each vertical tick mark on the x-axis represents results of cultures nasal lavages from a single pup. (TIF) [file ppat.1005887.s003.tif]

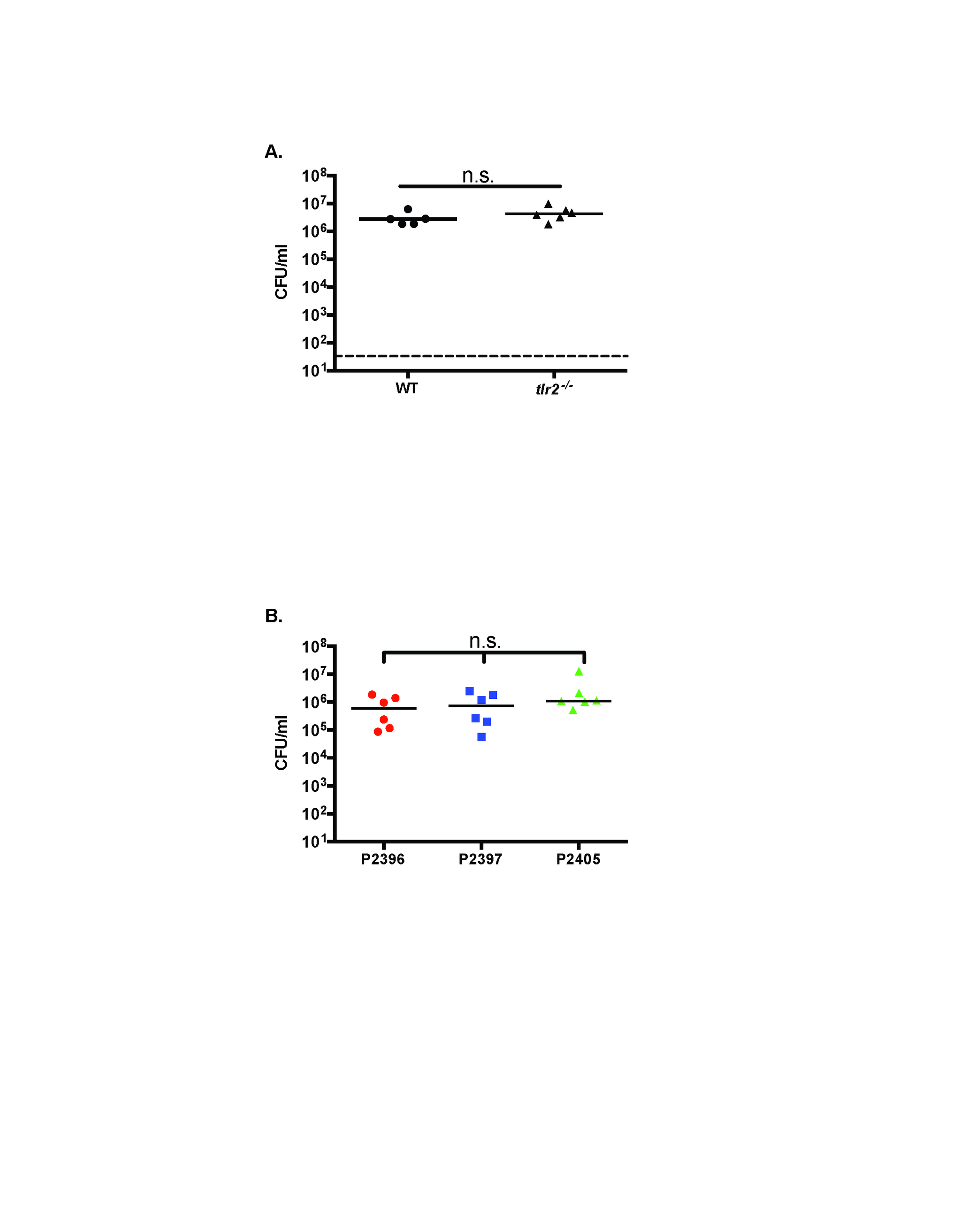

Supplement: S4 Fig — Pups were infected with S.pneumoniae on day 4 and IAV on day 8 of age. Nasal lavages were collected on day 12 of age and the density of colonized pneumococci quantified. A. Wildtype and tlr2 -/- pups infected with P1547. n.s. not significant (Mann-Whitney U test). B. tlr2 -/- index pups (n = 6) infected with an equal mixture of P2396, P2397 and P2405. Repeated measures (RM) one-way ANOVA test was used for statistical analysis. n.s. not significant. (TIF) [file ppat.1005887.s004.tif]
